# Editorial: The Roles of Checkpoint Inhibitors in Inflammatory Diseases

**DOI:** 10.3389/fimmu.2021.795495

**Published:** 2021-10-28

**Authors:** Andreas von Knethen, Jose-Ignacio Rodriguez-Barbosa

**Affiliations:** ^1^ Department of Anaesthesiology, Intensive Care Medicine and Pain Therapy, University Hospital Frankfurt, Frankfurt, Germany; ^2^ Transplantation Immunobiology, School of Biology and Biotechnology, Institute of Molecular Biology, Genomics and Proteomics, University of Leon, Leon, Spain

**Keywords:** sepsis, immune checkpoint inhibition, PD1-PD-L1, inflammation, tolerance

The engagement of inhibitory receptors in T cells by their corresponding ligands are crucial mediators for the maintenance of immune cell tolerance to self and for the modulation of the course of an immune response and the return to homeostasis, once the initial antigenic stimulus has been cleared (1). This is especially relevant in cancer treatment and inflammatory chronic diseases in which antigen persistence prevents the contraction phase of T cell differentiation leading to T cell dysfunction that promotes tumor escape or prevents immunopathology, respectively (2). Tumor cells co-opt these physiological mechanisms and upregulate ligands for co-inhibitory receptors, such as PD-L1, to defend themselves from immune attack (3, 4). Therefore, blockade of immune-checkpoints in cancer immunotherapy has become a new paradigm in the fight against cancer. However, the downside face of enhanced anti-tumor responses comes at the price of breaking tolerance to self and the induction of inflammation and life-threatening autoimmune diseases, two immune-related adverse effects (irAE) of immune-checkpoint inhibition (ICI) (5).

In line, considering skin inflammation as one of the main immune-related adverse effects (irAEs) of immune checkpoint blockade, Ashoori et al. point out in a mouse model of oxazolone-induced contact hypersensitivity that skin inflammation is aggravated following PD-L1 inactivation by a neutralizing antibody. This leads to skin CD8^+^ T-cell infiltration and subsequent inflammation, which is not evidenced in recombinase deficient (RAG2^-/-^) mice, where oxazolone-induced skin inflammatory ear swelling did not increase in response to PD-L1 inhibition. CD8^+^ T cell infiltration correlates with an increased expression of the chemokines CXCL9 and CXCL10 in non-immune cells (CD45^-^) of the inflamed ear, two cytokines produced in response to CD8 T cell activation and IFN-γ secretion.

To anticipate the potential involvement of co-inhibitory ligands in the regulation of tissue specific inflammatory T cell-mediated responses, the demonstration of expression of these ligands in the hematopoietic and non-hematopoietic microenvironment is required. Focusing on the kidney, Hakroush et al. determine in mouse models of ischemia/reperfusion (IRI), folic acid-induced nephropathy (FAN), unilateral ureteral obstruction (UUO), and nephrotoxic serum nephritis (NTN) expression of PD-L1. Linked to cellular damage in the kidney, PD-L1 is induced after IRI, FAN, and NTN but not following post-renal UUO. Depending on the kidney injury model, PD-L1 expression is upregulated in tubuli or glomeruli, respectively in a panel of 87 human renal samples, including nephrectomy control kidneys, renal biopsies derived from patients with ICI-related acute interstitial nephritis (AIN), as well as renal biopsies derived from patients without ICI treatment. Control kidneys are PD-L1 negative, whereas in ICI-treated patients, PD-L1 expression is observed. In non-ICI treated patients, 19 out of 68 show renal PD-L1 expression. Thus, renal PD-L1 expression might be a pre-requisite of AKI development following ICI therapy.

In a mouse model of ischemia-induced hind limb inflammation following femoral artery (FA) ligation, Liu et al. tested the hypothesis that genetic ablation or pharmacological inhibition of PD-1 impair skeletal muscle inflammation. In corroboration, PD-1 knockout increases immune cell infiltration and pro-inflammatory (IFN-γ, TNF-α) cytokine expression. These effects also occur when PD-1 blocking antibodies are used in wild type mice. The authors determine the role of IFN-γ in angiogenesis regulation *in vitro* in HUVECs, where IFN-γ stimulation suppresses migration, proliferation, and tube formation of HUVECs, pointing to a direct role of IFN-γ in regulating angiogenesis.

Signaling through co-inhibitory receptors in T cells also contributes to immunosuppression during sepsis (6–8). Considering these previous data, Fallon et al. analyze the survival and pulmonary injury after sepsis in murine and human neonates. Following intraperitoneal cecal slurry injection to induce sepsis in 5 to 7 days old mouse pups, PD-1 deficiency (PD-1^-/-^) improved survival up to 90% compared to wild type mice with only 52% survival, although PD-L1 knockout (PD-L1^-/-^) mice do not significantly showed improved survival compared to their wild type counterparts (70% *vs* 52%). Moreover, in lung tissue of human neonates, significantly more PD-1 expressing cells are detected in samples derived from newborns with an intrauterine infection compared to specimens without an infection.

With a mathematical modelling approach, Gillis et al. suggest a new optimization method for sepsis treatment. As exemplified with the combinatory use of the PD-1 specific antibody nivolumab and the antibiotic meropenem, this methodology can be used to assess the potency of a combined regime of antibiotics and immunotherapy. Extending the established setting (9), this model takes into account CTL exhaustion, T cell reactivation and PK/PD values of the used drugs.

Nakamori et al. summarize the beneficial effect of targeting of PD1/PD-L1 interaction to prevent immune paralysis in sepsis and septic shock. The authors point out that based on the complexity and heterogeneity of the disease, a uniform therapy concept is not appropriate for an effective therapy. Therefore, the stratification of sepsis patients into subgroups with a similar etiology and characteristics of disease progression with the support of artificial intelligence (AI) methodology, the outcome of disease treatment may improve and be more cost-effective than current approaches.

T cell exhaustion in sepsis is not limited to the immune checkpoint protein PD-1 and its ligand PD-L1 as outlined by McBride et al. Other co-inhibitory pathways have been proposed in pre-clinical and clinical studies as contributors of immune paralysis during sepsis. These data support the assumption that intervening with ICI signaling can be a novel concept, worth testing to prevent/correct immune paralysis in sepsis patients.

Pfeifhofer-Obermair et al. zoomed on iron as an important component for host-pathogen interactions. In a chronic mouse model of *Salmonella enterica* serovar *typhimurium* infection the negative immune checkpoint regulator T cell immunoglobulin and mucin-domain containing protein 3 (TIM-3) was upregulated following an iron rich diet compared to a low iron diet. Increased TIM-3 expression blocks the differentiation of Th1 IFN-γ^+^ cells, which is restored employing a TIM-3 neutralizing antibody, consequently improving immunity towards the pathogen in an iron-enriched environment.

The manuscripts collected in this Research Topic exemplify the important role of ICIs in inflammatory diseases. Obviously, this is a rapidly growing field, requiring further *in vitro* and *in vivo* studies to fully understand and consequently develop the potential of this therapy concept.

## Author Contributions

All authors contributed to this editorial insight and approved the submitted version.

## Funding

This research was supported by grants from the Deutsche Forschungsgemeinschaft (KN493/13-1 and SFB815 TP3). The Else Kröner-Fresenius Foundation (EKFS), the Research Training Group Translational Research Innovation-Pharma (TRIP), and the State of Hessen to the LOEWE Research Centre for Translational Medicine and Pharmacology in part funded the work. This work has been supported by grants of the Spanish Ministry of Science and Universities (Grant # PID2019-103984RB-I00), Department of Education of Castilla and Leon Regional Government (Grant # LE-006P20) to JIRB and it was also partially funded by the Spanish Network of Cancer Research, Ciberonc (Grant # CB16/12/00480).

## Conflict of Interest

The authors declare that the research was conducted in the absence of any commercial or financial relationships that could be construed as a potential conflict of interest.

## Publisher’s Note

All claims expressed in this article are solely those of the authors and do not necessarily represent those of their affiliated organizations, or those of the publisher, the editors and the reviewers. Any product that may be evaluated in this article, or claim that may be made by its manufacturer, is not guaranteed or endorsed by the publisher.

## References

[1] Keir ME Butte MJ Freeman GJ Sharpe AH. PD-1 and its Ligands in Tolerance and Immunity. Annu Rev Immunol (2008) 26:677–704. doi: 10.1146/annurev.immunol. 26.021607.090331. 18173375 PMC10637733

[2] Bagchi S Yuan R Engleman EG. Immune Checkpoint Inhibitors for the Treatment of Cancer: Clinical Impact and Mechanisms of Response and Resistance. Annu Rev Pathol (2021) 16:223–49. doi: 10.1146/annurev-pathol-042020-042741 33197221

[3] Liu J Chen Z Li Y Zhao W Wu J Zhang Z. PD-1/PD-L1 Checkpoint Inhibitors in Tumor Immunotherapy. Front Pharmacol (2021) 12:731798. doi: 10.3389/fphar.2021.731798 34539412 PMC8440961

[4] Wang Y Wang H Yao H Li C Fang J-Y Xu J. Regulation of PD-L1: Emerging Routes for Targeting Tumor Immune Evasion. Front Pharmacol (2018) 9:536. doi: 10.3389/fphar.2018.00536 29910728 PMC5992436

[5] Postow MA Sidlow R Hellmann MD. Immune-Related Adverse Events Associated With Immune Checkpoint Blockade. N Engl J Med (2018) 378:158–68. doi: 10.1056/NEJMra1703481 29320654

[6] Fallon EA Biron-Girard BM Chung C-S Lomas-Neira J Heffernan DS Monaghan SF. A Novel Role for Coinhibitory Receptors/Checkpoint Proteins in the Immunopathology of Sepsis. J Leukoc Biol (2018) 103:1151–64. doi: 10.1002/JLB.2MIR0917-377R PMC6314914 29393983

[7] Patil NK Guo Y Luan L Sherwood ER. Targeting Immune Cell Checkpoints During Sepsis. Int J Mol Sci (2017) 18:2413–37. doi: 10.3390/ijms18112413 PMC5713381 29135922

[8] Wakeley ME Gray CC Monaghan SF Heffernan DS Ayala A. Check Point Inhibitors and Their Role in Immunosuppression in Sepsis. Crit Care Clin (2020) 36:69–88. doi: 10.1016/j.ccc.2019.08.006 31733683 PMC6863093

[9] Gillis A Beil M Halevi-Tobias K van Heerden PV Sviri S Agur Z. Alleviation of Exhaustion-Induced Immunosuppression and Sepsis by Immune Checkpoint Blockers Sequentially Administered With Antibiotics-Analysis of a New Mathematical Model. Intensive Care Med Exp (2019) 7:32. doi: 10.1186/s40635-019-0260-3 31187301 PMC6560115

